# Principles and practice of the biologic therapy of cancer (third edition)

**DOI:** 10.1038/sj.bjc.6600090

**Published:** 2002-02-12

**Authors:** RTD Oliver

**Affiliations:** Medical Oncology Department, St Bartholomew's Hospital, 1st floor King George V Building, West Smithfield, London EC1A 7BE, UK

## Abstract

*British Journal of Cancer* (2002) **86**, 662–663. DOI: 10.1038/sj/bjc/6600090 www.bjcancer.com

© 2002 Cancer Research UK

## 

Edited by Steven A Rosenberg. Publishers: Lippincott Williams & Wilkins. ISBN 0-7817-2272-1. $149.00. 944 pages

Compared to the near Biblical reverence given to de Vita's large compendium Principles and Practice of Oncology, this 130 author, 64 chapter 897 page volume out of the same publishing stable is probably somewhat less known to most cancer doctors whether established or in training. As a life long devotee of ‘biotherapy’ during several of its peaks and troughs of interest over the last 25 years, I have to confess to having not read either its first or second editions from 1991 and did not have them available to compare with the third edition, of which I have just completed a detailed and highly educational read.

Mainstream oncologists treating the big four cancers, i.e. lung, breast, colon and prostate, might at first sight find little to excite them. This is because there have been so few studies of biotherapy in these fields because they provide such a dominant market for the palliative cytostatic chemotherapy and endocrine agents that rarely produce durable complete remission. That the future in breast and prostate cancer may be more interesting is raised by observations from a small breast cancer study in Japan. This highlighted the fact that until that study which reported a doubling of survival, none had examined biotherapy in combination with endocrine therapy in patients with hormone sensitive cancer. All previous reports from breast cancer were in patients treated after escape from hormone control.

Another notable absence from this book are any studies on combination between radiation and biotherapy apart from the section on use of radio nucleotides to enhance activity of monoclonal antibodies. This is clearly not due to omission, as there are no recent studies, but because this discipline, focused by the need to understand physics, has shown little interest in learning about this area of biology. With anecdotal reports of patients loosing feet from radiation induced arteritis after TNF limb perfusion treatment, dying of acute encephalitis after brain radiation while taking Interferon alpha and dying from exfoliative dermatitis after wide field low voltage radiation while taking interleukin-2, it is clear that bio therapeutic drugs are rather powerful radiation sensitizers and if used in smaller doses might improve local control. With considerable progress being made with computerized conformal radiation field control this could be an important area for the future in many fields including the common adult solid cancers such as lung, rectal and prostate cancer.

The book itself is a real gold mine of information albeit from an American perspective, covering all of the most significant studies of the last 15 years with data on more than 6000 patients in cytokine trials (12–26% response 2–5% for greater than 5–10 years), 750 in cell therapy trials (mostly not an improvement though recent trials with mini-allographs achieving 40% responses), 1400 in trials of monoclonal antibodies (11–46% response), 128 in gene therapy studies (mostly in progress though three reported an 8–22% response) and more than 300 patients measurable disease phase 2 studies of experimental tumour vaccines (21% responses) and as yet no data in a multiplicity of trials of antiangiogenic drugs now underway. By providing such extensive overview data, the need for more systematic analysis with longer follow up of these trials is becoming apparent. This is because late follow up is confirming the early indications of durable complete remissions that are long lasting with patients now regularly alive disease free for more than 10 years, albeit only 2–5% of the original cases. Moreover improved prognostication for response is helping to be more focused in patient selection. However this is one area where there could be even more improvement from advances in DNA, RNA and protein typing that has already provided such improved prognostication in other tumour sites. Success with such prognostication might also ultimately facilitate ways of identifying biotherapy sensitive minority of patients with the common cancers not currently treated with biotherapy.

While the cytokine chapters are dominated by studies in Melanoma and Renal Cell Cancer on interferon and interleukin-2 and the debates on whether high dose IL-2 is really better than low dose IL-2 or single agent interferon, they provide little discussion on the failure to improve on the results of monotherapy by combining agents as had been shown in animal studies. The chapters on IL-4, TNF, IL-12, gamma Interferon and gene therapy highlight additional areas where encouraging animal studies have failed to deliver clinical gain. However each of these chapters provides hope for new gains in the future. Most exciting of these undoubtedly is the chapter on Tumour necrosis factor alpha. Almost certainly this cytokine was the mediator of the minority of durable complete responses reported by Coley from use of bacterial endotoxin in the early part of the 19th century. Despite this, the initial results from TNF phase 1 studies were abysmal with 2.2% response in 566 patients treated in a multitude of different schedules. However reports from its use intraperitoneally to manage ascites in a series of 79 patients with 71% improvement, taken with results from those on 111 melanoma patients and 309 sarcoma treatment with intra-arterial limb perfusion with melaphalan and TNF (± gamma Interferon) with 79 and 33% local complete response provide a model that may be relevant for improving the results of local control of all solid cancer surgery and also radiotherapy if the anecdotal report of enhanced vascular damage proves generalizeable.

At first sight the chapters on gene therapy and genetically modified vaccines were equally disappointing to that on TNF particularly so given the amount of hype there has been over the area. However, one throw away line from the studies of intra-tumoural p53 in lung cancer provides an assurance that there could be more to come. This observation was the fact that in the trials of intratumoural injection of p53 in lung cancer there were post mortem studies showing complete remission in tumour injected via the bronchoscope. Furthermore, though the overall response rate in these studies was 20% in 25 cases and fell from 33% in the phase 1 to 13% in the phase 2, more significant was the fact that six of the 12 patients who had collapsed lung from bronchial obstruction had seen sufficient tumour shrinkage to enable re-expansion of the lung. Like the studies on TNF arterial perfusion this data is highlighting the potential for highly selective therapy to be focused on the primary tumour with the possibility of achieving durable complete remissions in a high proportion of patients and improving local control.

Two other areas of particular interest emerge from the extensive data brought together in this volume. The first relates to the treatment of follicular lymphoma. Despite this cancer having a reputation for indolence such that observation can occasionally be justified as primary management because of a spontaneous regression rate of 25% and also having one of the highest responses even after failed chemotherapy to interferon alpha (30–40%), to IL-2 (26%) and to monoclonal antibodies such as the B lymphocyte antigen anti CD20 (53%), the only first line studies are of its use in combination with chemotherapy.

The final area of interest raised by this book is the question of use of erythropoietin in cancer patients. While few can dispute the quality of life gain from reduction of tiredness on chemotherapy, it is unlikely to increase long term cure or be a routine for any but the most well funded services. Far more interesting and as yet unresolved is the question whether there is any place to justify use of this cytokine to treat the anaemia which is found in patients with advanced cancer to improve their chances of responding to standard treatment. There are two situations already providing strong justification for this thought. Firstly there are trials in cervix cancer patients undergoing treatment with radiation aimed to reduce tumour hypoxia. Secondly there is *in vitro* evidence that erythropoietin treatment of cancer cell lines can enhance killing by immune cells.

From this brief review it is clear that this book brings together a body of data that has accumulated from a wide range of sources that suggests biotherapy has a wider importance than is generally appreciated by physicians and surgeons involved in treatment of cancer patients. As a consequence there could be a case for this volume to be as much compulsory reading as its companion on Principles and Practice of Oncology for all trainees in fields involved in care of cancer patients.

